# *Aspergillus calidoustus* and *Talaromyces columbinus* infections in chronic graft-versus-host disease

**DOI:** 10.1007/s00277-024-05980-w

**Published:** 2024-09-04

**Authors:** Emanuele Pacini, Silke Schelenz, Alireza Abdolrasouli, Varun Mehra, M. Mansour Ceesay, Antonio Pagliuca, Daniele Avenoso

**Affiliations:** 1 Department of Haematological Medicine, King’s College Hospital NHS Foundation Trust, London, UK; 2 Faculty of Medicine and Surgery, University of Siena, Siena, Italy; 3 Infection Sciences, Department of Medical Microbiology, King’s College Hospital, London, UK

**Keywords:** GVHD, IFIs, Allogeneic stem cell transplant, A. calidoustus, T. Colombinus, BAL, Posaconazole-resistant

## Abstract

Advancements in allogeneic haematopoietic stem cell transplant (alloHSCT) procedures have improved patient outcomes over the last two decades, though invasive fungal infections (IFIs) remain a significant risk. The incidence of IFIs in alloHSCT recipients is estimated at 6%, with a mortality rate of 13%, and Aspergillus species are the most common pathogens involved. Posaconazole is effective in preventing IFIs post-transplant and is standard care during neutropenia or when managing graft-versus-host disease (GvHD) with high-dose steroids. However, azole prophylaxis may cause resistant Aspergillus species like A. calidoustus, which are difficult to treat. We report a case from our institution where a patient developed a dual infection with Aspergillus calidoustus and Talaromyces columbinus after alloHSCT and posaconazole prophylaxis. While A. calidoustus is known to cause IFIs in HSCT recipients, T. columbinus represents a previously unreported occurrence in medical literature. This case underscores the importance of a multifaceted diagnostic strategy, integrating BAL diagnosis, mycological cultures, direct microscopy, fungal speciation, susceptibility testing, and biomarkers. These comprehensive approaches are indispensable for accurate pathogen identification and effective management of IFIs with appropriate antifungal agents.

Advancements in allogeneic haematopoietic stem cell transplant (alloHSCT) procedures have improved patient outcomes over the last two decades, though invasive fungal infections (IFIs) remain a significant risk. The incidence of IFIs in alloHSCT recipients is estimated at 6%, with a mortality rate of 13%, and *Aspergillus species* are the most common pathogens involved [1]. Posaconazole is effective in preventing IFIs post-transplant [2] and is standard care during neutropenia or when managing graft-versus-host disease (GvHD) with high-dose steroids [3]. However, azole prophylaxis may cause resistant *Aspergillus species* like *A. calidoustus*, which are difficult to treat [4]. We report a case from our institution where a patient developed a dual infection with *Aspergillus calidoustus* and *Talaromyces columbinus* after alloHSCT and posaconazole prophylaxis. While *A. calidoustus* is known to cause IFIs in HSCT recipients, *T. columbinus* represents a previously unreported occurrence in medical literature.

A 30-year-old man with chronic myeloid leukaemia resistant to tyrosine kinase inhibitors underwent myeloablative haploidentical HSCT after conditioning consisted of thiotepa 10 mg/Kg, busulfan 9.6 mg/kg, fludarabine 150 mg/m^2^; GvHD prophylaxis was with post-transplant cyclophosphamide, micofenolate and tacrolimus as previously described by our group [5]. Neutrophil and platelet engraftment occurred on day + 14 and + 17, respectively. Despite initial successful treatment with steroids (prednisolone 1 mg/kg/day) and posaconazole for chronic GvHD of the skin and liver (diagnosed on day + 147), he developed severe lung GvHD on day + 240, necessitating prednisolone 0.5 mg/kg/day, tacrolimus 2 mg/kg/day, and posaconazole. His condition worsened following severe dyspnoea and haemoptysis, leading to his hospitalisation on day + 247. Results of serial serum fungal biomarkers are shown in Fig. 1A. High resolution computerised tomography of thorax showed discrete areas consolidation (not lobar) and concomitant serum galactomannan index was raised at 0.975. Bronchoalveolar lavage fluid galactomannan index was high at 5.2. Direct microscopic examination of BAL fluid showed two distinct types of hyphal structures (Fig. 1B) and both *A. calidoustus* and *T. columbinus* were isolated in culture and subsequently identified using Matrix-Assisted Laser Desorption/Ionization Time-of-Flight (MALDI-ToF) mass spectrometry (Bruker, Daltonics) and ITS-based sequencing. Dual antifungal therapy with anidulafungin and isavuconazole was initiated, based on antifungal susceptibility data (Fig. 1C), and previous experiences by other groups [6–9], and additional GvHD treatment with extracorporeal photopheresis. Despite these efforts, the patient’s condition deteriorated, developed respiratory insufficiency and succumbed to his complications.

**Fig. 1:**
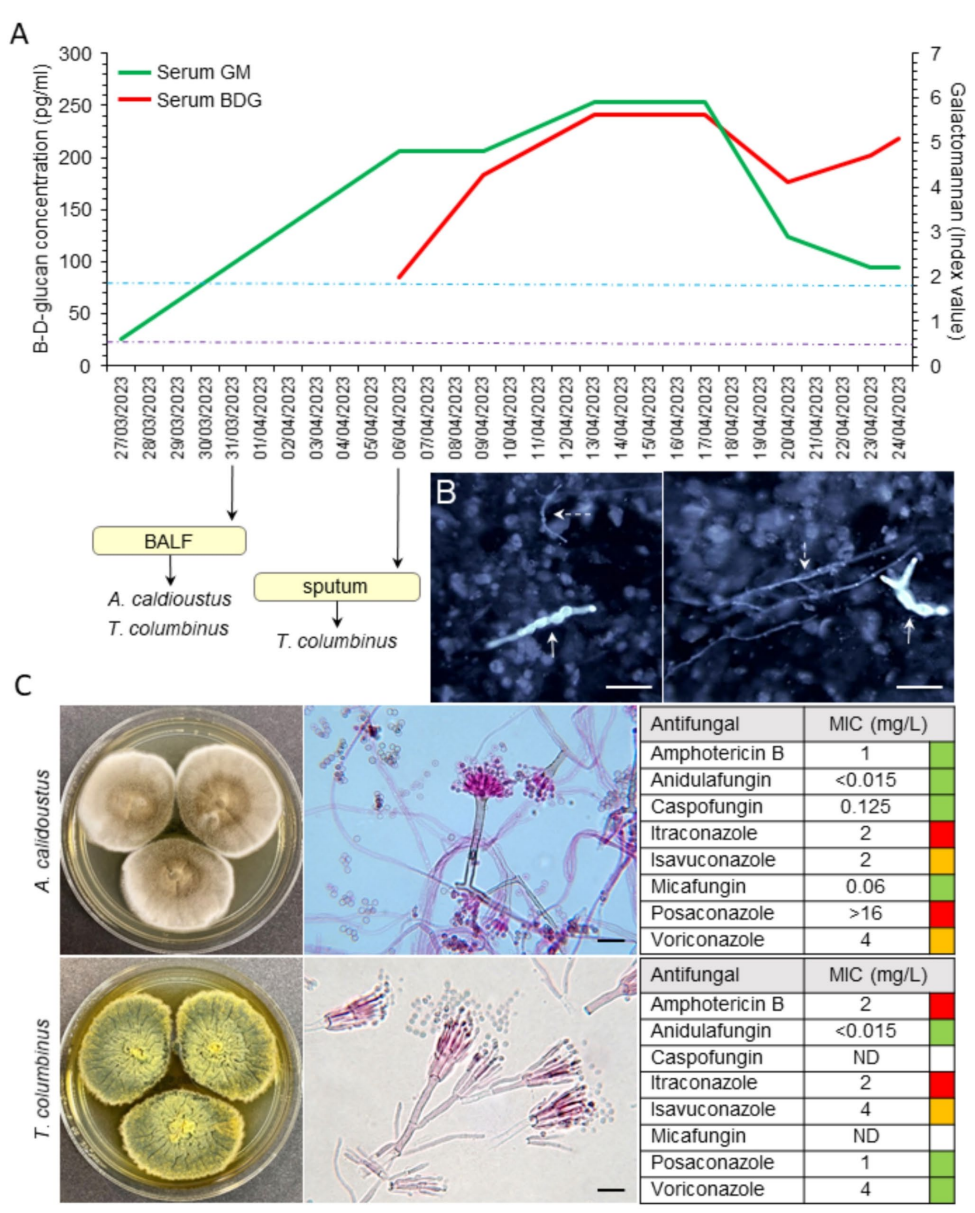
**(A)** Serial measurements of serum β-D-glucan and galactomannan over time with indication when respiratory samples were collected, **(B)** Direct microscopic examination of bronchoalveolar lavage (BAL) fluid using Calcofluor-white stain. Solid white arrows show *Aspergillus* hyphal fragments and dashed white arrows demonstrated fungal hyphae that belong to *Talaromyces* species. Both hyphae are septated, however *Talaromyces* hyphae are narrower when compared to *Aspergillus* species. **(C)** Macroscopic colony features and microscopic characteristics of both isolates with their corresponding antifungal susceptibility profiles using standard CLSI broth microdilution method. Green: sensitive, orange: intermediate, and red: resistant. (BALF: bronchoalveolar lavage fluid, BDG: β-D-glucan, GM: galactomannan, MIC: minimum inhibitory concentration, ND: not done, scale bar = 10 μm)

The identification of *Talaromyces columbinus* and *Aspergillus calidoustus* from BAL fluid, rather than sputum alone, underscores the importance of multiple diagnostic specimens for accurate pathogen isolation and identification. Direct fungal microscopy, fungal speciation, and susceptibility testing enabled rapid diagnosis of IFI, precise identification, and informed the selection of dual antifungal therapy with anidulafungin and isavuconazole, which was based on susceptibility data. This case highlights the critical link between thorough diagnostic work up and effective treatment strategies in managing IFIs in immunocompromised patients. Additionally, the use of fungal biomarkers (BD-glucan and galactomannan) provided data on infection dynamics and treatment response, as shown in Fig. 1A.

In conclusion, this case underscores the importance of a multifaceted diagnostic strategy, integrating BAL diagnosis, mycological cultures, direct microscopy, fungal speciation, susceptibility testing, and biomarkers. These comprehensive approaches are indispensable for accurate pathogen identification and effective management of IFIs with appropriate antifungal agents, particularly in complex and resistant cases. This case could represent an isolated event, as it is the first report of T. columbinus co-infection with azole-resistant Aspergillus species post-HSCT; however, it highlights the current unmet needs of finding new therapeutic agents against pathogenic species resistant to the antifungal drugs available and the development of effective prophylactic measures against pathogens not sensitive to posaconazole.

## Data Availability

No datasets were generated or analysed during the current study.
